# Potentially Heterogeneous Cross-Sectional Associations of Seafood Consumption with Diabetes and Glycemia in Urban South Asia

**DOI:** 10.3390/ijerph17020459

**Published:** 2020-01-10

**Authors:** Matthew O. Gribble, Jennifer R. Head, Dorairaj Prabhakaran, Deksha Kapoor, Vandana Garg, Deepa Mohan, Ranjit Mohan Anjana, Viswanathan Mohan, Sudha Vasudevan, M. Masood Kadir, Nikhil Tandon, K. M. Venkat Narayan, Shivani A. Patel, Lindsay M. Jaacks

**Affiliations:** 1 Department of Environmental Health, Emory University Rollins School of Public Health, Atlanta, GA 30322, USA; 2 Public Health Foundation of India, Gurgaon Haryana 122002, India; 3 All India Institutes of Medical Sciences, Delhi 110029, India; 4 Madras Diabetes Research Foundation, Chennai 600086, India; 5 Department of Community Health Sciences, Aga Khan University, Karachi 74800, Pakistan; 6 Hubert Department of Global Health, Emory University Rollins School of Public Health, Emory Global Diabetes Research Center, Atlanta, GA 30322, USA; 7 Department of Global Health and Population, Harvard University T.H. Chan School of Public Health, Boston, MA 02115, USA

**Keywords:** diabetes mellitus, glycated hemoglobin A, blood glucose, seafood, shellfish, diet, diet surveys, India, Pakistan

## Abstract

*Aims*: In this study, we aimed to estimate cross-sectional associations of fish or shellfish consumption with diabetes and glycemia in three South Asian mega-cities. *Methods*: We analyzed baseline data from 2010–2011 of a cohort (*n* = 16,287) representing the population ≥20 years old that was neither pregnant nor on bedrest from Karachi (unweighted *n* = 4017), Delhi (unweighted *n* = 5364), and Chennai (unweighted *n* = 6906). Diabetes was defined as self-reported physician-diagnosed diabetes, fasting plasma glucose ≥126 mg/dL (7.0 mmol/L), or glycated hemoglobin A1c (HbA1c) ≥6.5% (48 mmol/mol). We estimated adjusted and unadjusted odds ratios for diabetes using survey estimation logistic regression for each city, and differences in glucose and HbA1c using survey estimation linear regression for each city. Adjusted models controlled for age, gender, body mass index, waist–height ratio, sedentary lifestyle, educational attainment, tobacco use, an unhealthy diet index score, income, self-reported physician diagnosis of high blood pressure, and self-reported physician diagnosis of high cholesterol. *Results*: The prevalence of diabetes was 26.7% (95% confidence interval: 24.8, 28.6) in Chennai, 36.7% (32.9, 40.5) in Delhi, and 24.3% (22.0, 26.6) in Karachi. Fish and shellfish were consumed more frequently in Chennai than in the other two cities. In Chennai, the adjusted odds ratio for diabetes, comparing more than weekly vs. less than weekly fish consumption, was 0.81 (0.61, 1.08); in Delhi, it was 1.18 (0.87, 1.58), and, in Karachi, it was 1.30 (0.94, 1.80). In Chennai, the adjusted odds ratio of prevalent diabetes among persons consuming shellfish more than weekly versus less than weekly was 1.08 (95% CI: 0.90, 1.30); in Delhi, it was 1.35 (0.90, 2.01), and, in Karachi, it was 1.68 (0.98, 2.86). *Conclusions*: Both the direction and the magnitude of association between seafood consumption and glycemia may vary by city. Further investigation into specific locally consumed seafoods and their prospective associations with incident diabetes and related pathophysiology are warranted.

## 1. Background

Studies of the association between seafood consumption and diabetes risk were equivocal and reported geographically heterogeneous results. A 2012 systematic review of 18 prospective cohorts observed lower risk with increasing seafood consumption in studies conducted in East Asia (Japan and China), but increased risk in studies conducted in North America or Europe [1]. Among individuals with diabetes, the effect of seafood consumption on glycemic control is similarly unclear. A 2008 Cochrane review of 23 randomized trials conducted among persons with diabetes found no overall effect of omega-3 supplements on glycemic control or fasting insulin [2]. These findings suggest that pooled associations may not be suitable for locally appropriate nutritional messaging.

The geographic heterogeneity of associations between fish consumption and diabetes might reflect the extensive spatial variation in nutrient and contaminant composition of fish [3,4,5,6,7]. Some of these contaminants, particularly organochlorine pesticides such as hexachlorocyclohexane (HCH), were implicated as risk factors for diabetes [8]. India, which witnessed an unprecedented increase in diabetes over the past two decades, was noted as the country most contaminated by HCH in 1990 [9]. In a 2004 market survey in Coimbatore, India, 22% of the sampled commercial marine fishes had levels of HCH above the maximum residue limits set by the Food and Agriculture Organization of the United Nations and World Health Organization (FAO/WHO) for fish products [10]. Other organohalogen contaminants were also detected above safe levels in some Indian fish [11], although other surveys found safe levels of contaminants in other fish [12,13,14]; thus, there is some heterogeneity in risks posed by different fish. In Pakistan, some studies suggest possible fish contamination by organochlorines including HCH [15,16], although other fish have low enough concentrations to pose limited health risks [17]. There is also evidence that shellfish (e.g., mussels) from tributaries of the river Ravi in Pakistan could be contaminated by HCH and other organohalogens above safe levels [18]. Fish on the Karachi coast had levels of HCH of 5.85 μg/g lipid detected [19]. Providing meaningful nutritional guidance on the value of seafood for diabetes prevention and management for South Asian populations may require examination of associations at smaller spatial scales.

## 2. Aims

The objective of this study was to examine the cross-sectional, local associations of seafood consumption with diabetes prevalence and glycemia in three geographically varied South Asian megacities: two on the coast of the Indian Ocean (Chennai on the Bay of Bengal, Karachi on the Arabian Sea) and one inland (Delhi).

## 3. Methods

### 3.1. Study Population

The Cardiometabolic Risk Reduction in South Asians (CARRS) study is an ongoing, population-based cohort study drawn from complex survey samples of three major cities: Karachi, Delhi, and Chennai [20]. The inclusion criteria were that participants were ≥20 years old and a permanent resident of a selected household; the exclusion criteria for participants were pregnancy or bedrest. Survey estimation based on this sample is, therefore, representative of the adult residential population of these cities without pregnancy or bedrest. Here, we use data from the 2010–2011 CARRS baseline visit. All procedures were approved by the ethics review committees at Emory University (IRB00044159), Public Health Foundation of India (IRB00006658), All India Institute of Medical Sciences (IEC/NP-17/07.09.09), Aga Khan University (1468-CHS-ERC-2010), and Madras Diabetes Research Foundation (IRB00002639). Written informed consent was obtained from all participants.

### 3.2. Outcome Measures

Fasting plasma glucose was measured using the hexokinase/kinetic method in Chennai and Delhi and the glucose oxidase/endpoint method in Karachi. Glycated hemoglobin A1c (HbA1c) was measured using high-performance liquid chromatography at all three sites. Diabetes was defined as self-reported physician-diagnosed diabetes, fasting plasma glucose ≥126 mg/dL (7.0 mmol/L), or HbA1c ≥6.5% (48 mmol/mol) [21].

### 3.3. Dietary Data

Dietary data were obtained from a food propensity questionnaire modeled after the INTERHEART case–control study food frequency questionnaire [22,23]. The exposure variable was self-reported fish consumption in the past year (categorized as less than weekly (i.e., monthly or never), weekly, or more than weekly), and shellfish consumption in the past year (categorized as less than weekly, weekly, or more than weekly). Because overall diet quality is an important predictor of diabetes prevalence [24] and glycemia [25], we calculated an unhealthy diet score based on the consumption frequency of 10 food groups: meats, organ meats, desserts, deep-fried Western-style foods, deep-fried South Asian-style foods, Western-style desserts, South Asian-style desserts, refined cereals (boiled rice, fried rice, biryani, pulao, idli, dosa, semolina, sago, pearl barley, pasta, sheermal, taftan, and white bread), pickles, and carbonated beverages and other soft drinks. Participants were assigned a value of zero (never consume), one (consume monthly), two (consume weekly), or three (consume daily) for each of the 10 food groups, and the scores were summed such that the maximum unhealthy diet score was 30 and the minimum was zero.

### 3.4. Sociodemographic, Behavioral, and Anthropometric Data

Sociodemographic characteristics including age, gender, educational attainment, and monthly household income, as well as behavioral risk factors including tobacco and alcohol use, were assessed by a questionnaire administered by trained study staff. Questions were derived from questionnaires used in the Chennai Urban Population Study, the Chennai Urban Rural Epidemiological Study, and the Sentinel Surveillance Study [20]. Time spent sitting was estimated using the short form of the International Physical Activity Questionnaire [26].

Standardized procedures were used to measure weight to the nearest 0.1 kg (electronic body-composition analyzer, Tanita BC-418 in Delhi and Chennai, and Tanita BC-545 in Karachi, Tanita Co, Tokyo, Japan), height to the nearest 0.1 cm (portable stadiometer, SECA Model 213, SecaGmbH Co, Hamburg, Germany), and waist and hip circumferences to the nearest 0.1 cm (non-stretch measuring tape, Gulick II, Country Technology, Gays Mills, WI, USA) based on the United States (U.S.) National Health and Nutrition Examination Survey (NHANES-III) protocol [27]. Body mass index (BMI) was calculated as weight (kg) divided by height squared (m^2^).

### 3.5. Statistical Analysis

We estimated the means of continuous variables and proportions of categorical variables in the target populations of adults in Karachi, Chennai, and Delhi, according to the consumption of seafood. We estimated adjusted odds ratios for prevalent diabetes using logistic regression models, and adjusted differences in mean HbA1c and mean fasting glucose using linear regression models. All analyses used survey estimation techniques and appropriate weighting allowing for population inference. Confounders considered in regression analyses included participant age (linear and quadratic terms), masculine or feminine gender (one non-binary gender participant was excluded), measured BMI (<23 kg/m^2^, ≥23 and <25 kg/m^2^, ≥25 and <30 kg/m^2^, or ≥30 kg/m^2^), waist–height ratio, educational attainment (up to primary, high school/secondary, or college graduate), tobacco use (current, ever, or never), monthly household income (Indian rupees (INR) ≤ 10,000, equivalent to U.S. $200), INR 10,001–20,000 (U.S. $200–400), INR ≥ 20,001 (U.S. $400), or income not declared), tertile of unhealthy diet score, sedentary lifestyle (indicator for sitting or reclining but not sleeping ≥5 h/day), self-reported physician diagnosis of high blood pressure (yes or no), and self-reported physician diagnosis of high cholesterol (yes or no).

Missing data were handled by multiple imputation by chained equations [28]. The one transgender participant’s gender was recoded as missing to stabilize estimation (i.e., the analysis treated gender as a binary variable). Prior to imputation, *n* = 3756 participants in the sample (23%) were missing data on weight, *n* = 3650 (22%) missing data on waist–height ratio, *n* = 3515 (22%) missing data on height, *n* = 2655 (16%) missing data on HbA1c, *n* = 2568 (16%) missing data on fasting glucose, *n* = 2553 (16%) missing data on diabetes status, *n* = 2 (<1%) missing data on educational attainment, *n* = 2 (<1%) missing data on tobacco consumption, *n* = 1 (<1%) missing data on sedentary lifestyle, *n* = 1 (<1%) missing data on unhealthy diet score, *n* = 1 (<1%) missing data on fruit consumption, *n* = 1 (<1%) missing data on vegetable consumption, *n* = 1 (<1%) missing data on fish consumption, and *n* = 1 (<1%) missing data on shellfish consumption. Missing data were imputed by multiple imputation by chained equations considering gender, city, unhealthy diet score, consumption of fruits, consumption of vegetables, income category, sedentary lifestyle, fish consumption, shellfish consumption, age, weight, height, HbA1c, fasting glucose, diabetes status, educational attainment, tobacco consumption, waist–height ratio, self-reported physician diagnosis of high blood pressure, and self-reported physician diagnosis of high cholesterol. We imputed 70 datasets.

Because the types of seafood, likely nutrients and contaminants in the seafood, and confounder distributions vary geographically, all analyses were stratified by city (i.e., we provide survey subpopulation estimates). In a sensitivity analysis, due to the small number of shellfish-consuming women in Delhi, we examined associations of shellfish with outcomes restricted to men only in Delhi (i.e., survey subpopulation). In sensitivity analyses for the role of diet in shaping our results (Tables S1–S3, Supplementary Materials), we considered models unadjusted for unhealthy diet score, or adjusted instead for daily consumption of fruits and of vegetables. In sensitivity analyses for the role of body fat distribution (Tables S4 and S5, Supplementary Materials), we considered models unadjusted for waist–height ratio.

Analyses were conducted using Stata SE 14.2 (StataCorp LLC, College Station, TX, USA).

## 4. Results

### 4.1. Seafood Consumption by City

Demographic characteristics of the cities were summarized in subgroups defined by fish consumption frequency (Table 1) and by shellfish consumption frequency (Table 2). In Chennai, an estimated 72% (95% confidence interval (CI): 69%, 75%) of the population consumed fish more than weekly (in Delhi 10% (95% CI: 8%, 12%), in Karachi 12% (95% CI: 10%, 15%)), and, in Chennai, only an estimated 48% (95% CI: 45%, 50%) of the population consumed shellfish less than weekly. In Delhi, an estimated 95% (95% CI: 93%, 96%) of the population consumed shellfish less than weekly. In Delhi, there was less frequent shellfish consumption among women than men; an estimated 99% (95% CI: 98%, 99%) of women in Delhi consumed shellfish less than once per week, whereas an estimated 91% (95% CI: 88%, 93%) of men from Delhi consumed shellfish less than once per week. In Karachi, an estimated 91% (95% CI: 89%, 94%) of the population consumed shellfish less than weekly.

### 4.2. Seafood Consumption and Diabetes

The estimated prevalence of diabetes in Chennai was 33% (95% CI: 28%, 38%) among persons consuming fish less than weekly, 26% (95% CI: 22%, 30%) among persons consuming fish weekly, and 26% (95% CI: 23%, 29%) among persons consuming fish more than weekly. The estimated prevalence of diabetes in Delhi was 38% (95% CI: 34%, 42%) among persons consuming fish less than weekly, 32% (95% CI: 28%, 36%) among persons consuming fish weekly, and 37% (95% CI: 30%, 43%) among persons consuming fish more than weekly. The estimated prevalence of diabetes in Karachi was 24% (95% CI: 21%, 26%) among persons consuming fish less than weekly, 24% (95% CI: 21%, 27%) among persons consuming fish weekly, and 27% (95% CI: 22%, 33%) among persons consuming fish more than weekly.

The estimated prevalence of diabetes in Chennai was 27% (95% CI: 25%, 29%) among persons consuming shellfish less than weekly, 26% (95% CI: 24%, 29%) among persons consuming shellfish weekly, and 26% (95% CI: 23%, 29%) among persons consuming shellfish more than weekly. The estimated prevalence of diabetes in Delhi was 37% (95% CI: 33%, 41%) among persons consuming shellfish less than weekly, 36% (95% CI: 25%, 46%) among persons consuming shellfish weekly, and 42% (95% CI: 33%, 50%) among persons consuming shellfish more than weekly. The estimated prevalence of diabetes in Karachi was 24% (95% CI: 22%, 26%) among persons consuming shellfish less than weekly, 28% (95% CI: 21%, 34%) among persons consuming shellfish weekly, and 32% (95% CI: 22%, 41%) among persons consuming shellfish more than weekly.

Results from survey estimation logistic regression models are shown in Table 3. The unadjusted prevalence odds ratios for diabetes with more than weekly vs. less than weekly fish consumption were 0.72 (95% CI: 0.54, 0.96) in Chennai, 0.95 (95% CI: 0.72, 1.24) in Delhi, and 1.22 (95% CI: 0.92, 1.61) in Karachi. The adjusted prevalence odds ratios for diabetes for more than weekly vs. less than weekly fish consumption were 0.81 (95% CI: 0.61, 1.08) in Chennai, 1.18 (95% CI: 0.87, 1.58) in Delhi, and 1.30 (95% CI: 0.94, 1.80) in Karachi. The unadjusted prevalence odds ratios for diabetes comparing more than weekly vs. less than weekly shellfish consumption were 0.92 (95% CI: 0.78, 1.09) in Chennai, 1.23 (95% CI: 0.84, 1.81) in Delhi, and 1.48 (95% CI: 0.94, 2.33) in Karachi. In adjusted models, the prevalence odds ratios for more than weekly vs. less than weekly shellfish consumption were 1.08 (95% CI: 0.90, 1.30) in Chennai, 1.35 (95% CI: 0.90, 2.01) in Delhi, and 1.68 (95% CI: 0.98, 2.86) in Karachi. In the sensitivity analysis restricting to men in Delhi, results were still null; the adjusted odds ratio of prevalent diabetes comparing weekly vs. less than weekly shellfish consumption among men was 0.92 (95% CI: 0.66, 1.27), and the odds ratio of prevalent diabetes comparing more than weekly vs. less than weekly shellfish consumption among men was 1.06 (95% CI: 0.78, 1.44). Results were robust to sensitivity analyses for the influence of dietary adjustment (Table S1, Supplementary Materials) and weight–height ratio adjustment (Table S4, Supplementary Materials).

### 4.3. Seafood Consumption and Glycemia

The confounder-adjusted associations of seafood consumption with fasting glucose suggested similar geographic heterogeneity (Table 4). The adjusted difference in fasting glucose comparing more than weekly vs. less than weekly fish consumption was −1.29 (95% CI: −5.58, +2.99) mg/dL in Chennai, +4.56 (−0.60, +9.72) mg/dL in Delhi, and +5.00 (95% CI: +0.68, +9.32) mg/dL in Karachi. More than weekly vs. less than weekly shellfish consumption in Chennai was associated with +2.15 mg/dL (95% CI: −1.69, +5.99) fasting glucose in the multivariable-adjusted model; in Delhi, the association was +1.14 (95% CI: −7.06, +9.34) mg/dL fasting glucose, and, in Karachi, the association was +6.08 (−2.71, 14.86) mg/dL fasting glucose. In the men-only sensitivity analysis for Delhi, results were still null; weekly vs. less than weekly shellfish consumption was associated with a −1.53 (95% CI: −10.73, 7.68) mg/dL difference in fasting glucose, and more than weekly vs. less than weekly shellfish consumption was associated with +2.18 (95% CI: −6.12, +10.48) mg/dL fasting glucose. Results were robust to sensitivity analyses for the influence of dietary adjustment (Table S2, Supplementary Materials) and weight–height ratio adjustment (Table S5, Supplementary Materials) The nominal association of fish with higher fasting glucose in Karachi remained nominally significant in sensitivity models adjusted for fruit and vegetable consumption instead of our unhealthy diet score index, but was not significant in models unadjusted for diet; however, the point estimates for association were similar across the three models.

Possible geographic heterogeneity in associations was likewise observed with HbA1c (Table 4). There was a difference of −0.06 (95% CI: −0.19, +0.07) in percent HbA1c in Chennai, +0.07 (−0.13, +0.28) in Delhi, and +0.10 (95% CI: −0.06, +0.26) in Karachi, comparing more than weekly vs. less than weekly fish consumption. In Chennai, the adjusted difference in percent HbA1c with more than weekly vs. less than weekly shellfish consumption was +0.11 (95% CI: −0.02, +0.24). The adjusted association of more than weekly shellfish vs. less than weekly shellfish consumption with HbA1c in Delhi was +0.05 (−0.22, 0.33) percent HbA1c. In Karachi, the association of more than weekly shellfish vs. less than weekly shellfish consumption with HbA1c was +0.14 (−0.12, +0.40) percent HbA1c. In the sensitivity analysis looking at men in Delhi, the results were still null; weekly vs. less than weekly shellfish consumption was associated with −0.08 (95% CI: −0.41, +0.24) difference in percent HbA1c; more than weekly vs. less than weekly shellfish consumption was associated with a +0.06 (95% CI: −0.25, +0.37) difference in percent HbA1c. Results were robust to sensitivity analyses for the influence of dietary adjustment (Table S3, Supplementary Materials) and weight–height ratio adjustment (Table S5, Supplementary Materials).

## 5. Discussion

This is among the first examinations of the prevalence of seafood consumption across major cities, and the association of seafood consumption with diabetes and glycemia in South Asia. We are aware of a few smaller surveys of seafood consumption in Karachi (*n* = 872) [29], Chennai (*n* = 2527) [30], and Kolkata, India (*n* = 701) [31], but these did not look at relationships of fish or shellfish specifically as exposures predictive of diabetes. Our estimates of associations of seafood with diabetes, although not statistically significant, are consistent with geographically heterogeneous relationships in urban South Asia. In Chennai, frequent fish consumption was suggestively associated with lower prevalence of diabetes, lower fasting glucose, and lower HbA1c, whereas, in Karachi, frequent fish consumption was suggestively associated with higher prevalence of diabetes, higher fasting glucose, and higher HbA1c. In general, these results were not statistically significant, although the association of fish consumption with fasting glucose was nominally significant in Karachi in the main analysis, and point estimates were robust in sensitivity analyses.

One previous nationally representative, population-based study in India found that the odds of self-reported diabetes were two times higher (odds ratio (95% CI): 2.02 (1.59, 2.57)) among adults consuming fish daily compared to those who never consumed fish [32]. Although this might reflect common but unhealthy fish preparation practices (e.g., frying), previous studies explicitly evaluating fried fish and diabetes and glycemia were equivocal [33,34]. The heterogeneity of associations between Chennai and Delhi in our study suggests that nation-level associations for all of India may be inadequate for developing appropriate dietary guidelines for cardiometabolic disease prevention in specific settings.

Although the cross-sectional observational data limit causal interpretations, we believe that differences between cities in the sources and specific types of seafood might explain some of the underlying differences in association seen in our analysis; variation between persons within cities could also contribute to some of the uncertainty of our estimates of association. Additional research, for example, a survey measuring the nutrients and contaminants in the fish and shellfish commonly purchased in each of these cities, is needed. It would also be beneficial if biomarker data could be collected on the population of human seafood consumers in these cities regarding their internal exposure to organohalogen contaminants and omega-3 fatty acids, to help quantify how much organohalogen exposure and omega-3 intake might result from fish and other sources. It would then be possible to also consider how important fish is as one source of exposure to toxicants alongside other potential sources, including other dietary components [35,36]. Detailed information on consumed seafood nutrient and contaminant loads, as well as other dietary component nutrient and contaminant contributions, could help elucidate which chemicals might mediate possible associations to distal health outcomes.

Our findings of geographic heterogeneity across South Asian megacities may suggest that meta-analysis of omega-3 supplementation or fish consumption benefits [2], or even regionally stratified meta-analysis [1] may fail to adequately capture the local context of appropriate seafood nutritional guidance. In the U.S. and Canada, when a local fish is known to be heavily contaminated with a toxic chemical, a “fish advisory” is typically issued to discourage local consumption [37,38]. Similar principles of contextually dependent risk–benefit analysis for seafood consumption might be able to inform nutritional epidemiology in other settings.

This study has numerous strengths, but several limitations. It is a large complex survey sample, ensuring reasonable inference for the populations of Karachi, Chennai, and Delhi. It uses standard protocols for data collection across the three sites and, therefore, the results are more directly comparable across locations than if different protocols were used in each place. It covers a broad geographic and cultural landscape with varying dietary traditions, ecosystems, and local sources of pollution. A major limitation of this study is that the data available on fish or shellfish consumption were quite coarse, lacking detail on the species of seafood consumed, the method of preparation, and the quantity. It is likely that the resulting measurement error could have contributed to limited estimation precision (e.g., wide confidence intervals) and the lack of significance of these findings; future studies should collect data on consumption of specific species. Commercially available seafood species in India are known to vary in their contamination by HCH and other pollutants [10]. This likely heterogeneity of the chemical composition of seafood items violates a standard assumption required for interpretable epidemiological comparisons, called the consistency assumption or the treatment–variation irrelevance assumption [39,40]. Although it is plausible that, qualitatively, different fish are consumed in Chennai than in Karachi, additional research is needed on the kinds of seafood consumed in each locale, and the chemical burdens thereof, to understand why fish consumption may be beneficial in Chennai and not in Karachi for diabetes. Another limitation is that there were missing data on a number of variables used in the analysis, which were imputed using a multiple imputation model. It is possible that, if the imputation model was mis-specified, it could contribute to information bias and residual confounding, even as it obviated a source of selection bias. This is a cross-sectional observational study and, therefore, it cannot distinguish reverse causation and may be vulnerable to unmeasured confounding. It is possible that having diabetes could cause a person to change their diet to incorporate additional fish in Chennai, but to substitute alternatives to fish in Karachi. Additional research including prospective studies with more detailed data on seafood consumption are needed to assess these intriguing relationships.

## 6. Conclusions

This cross-sectional observational study was suggestive but inconclusive regarding whether there are geographically heterogeneous associations between fish or shellfish consumption and diabetes-related outcomes. Further research, particularly prospective studies focused on more specific types of seafood, is needed.

## Figures and Tables

**Table 1:** Estimated population characteristics, by city and fish consumption. Survey estimation techniques were used to obtain estimates of population means and proportions. Here, the category “less than weekly” indicates that fish is consumed monthly or never. Disjoint categories within a column, within each city, sum to 100% (with rounding). Statistics are estimates for the characteristics of each city’s population of permanent residents age ≥20 years old, who are neither pregnant nor on bedrest. Population parameter estimates are based on a large survey sample including participants from Karachi (unweighted *n* = 4017), Delhi (unweighted *n* = 5364), and Chennai (unweighted *n* = 6906). U.S.—United States; INR—Indian rupee.

Characteristic	Fish Consumption (Estimates and 95% Confidence Intervals)		
	Less than Weekly 41.3% (36.1%, 46.5%)	Weekly 23.0% (21.0%, 24.9%)	More than Weekly 35.7% (30.4%, 41.0%)
**CHENNAI**			
Age: mean years	42.1 (40.5, 43.7)	41.0 (39.8, 42.3)	39.6 (38.7, 40.4)
Masculine gender: %	43.8 (40.3, 47.3)	45.3 (42.7, 47.8)	45.9 (44.8, 46.9)
Education: %			
Up to primary school	17.0 (11.9, 22.0)	16.8 (14.2, 19.5)	15.4 (13.4, 17.4)
High school/secondary	63.3 (58.0, 68.5)	69.5 (65.6, 73.4)	73.6 (71.0, 76.2)
College graduate	19.8 (16.1, 23.4)	13.7 (10.0, 17.5)	11.0 (9.4, 12.6)
Household income: %			
≤INR 10,000 (U.S. $200)	75.3 (70.3, 80.2)	80.8 (76.9, 84.7)	82.4 (79.6, 85.3)
INR 10,001–20,000	15.4 (11.7, 19.0)	14.5 (11.5, 17.6)	13.5 (11.0, 15.9)
≥INR 20,001 (U.S. $400)	8.0 (4.8, 11.3)	4.5 (2.5, 6.4)	3.7 (2.3, 5.0)
Declined to state income	1.3 (0.2, 2.5)	0.2 (<0.1, 0.5)	0.4 (0.2, 0.7)
Sedentary Lifestyle: %			
Sitting < 5 h/day	24.7 (18.9, 30.5)	28.0 (19.7, 36.4)	23.1 (17.3, 28.9)
Sitting ≥ 5 h/day	75.3 (69.5, 81.1)	72.0 (63.6, 80.3)	76.9 (71.1, 82.7)
Body mass index: mean kg/m^2^	25.5 (25.1, 25.9)	25.3 (24.8, 25.8)	25.6 (25.5, 25.8)
Unhealthy diet score: mean	8.5 (8.1, 8.9)	9.1 (8.5, 9.7)	9.6 (9.3, 9.9)
Systolic blood pressure: mean mm Hg	121.6 (119.9, 123.3)	121.3 (119.5, 123.1)	120.6 (119.1, 122.2)
Tobacco use: %			
Never smoker	83.5 (80.2, 86.7)	79.7 (76.9, 82.5)	76.3 (74.8, 77.7)
Former smoker (>6 months)	1.9 (0.9, 2.9)	2.0 (1.1, 3.0)	1.9 (1.4, 2.4)
Recent smoker (≤6 months)	14.6 (11.4, 17.9)	18.2 (15.4, 21.0)	21.8 (20.4, 23.2)
Diagnosed hyperlipidemia: %	2.2 (0.8, 3.7)	1.1 (0.4, 1.8)	1.6 (1.1, 2.2)
Diagnosed hypertension: %	14.7 (10.9, 18.5)	11.0 (8.5, 13.4)	9.5 (8.0, 10.9)
**DELHI**			
Age: mean years	43.3 (41.5, 45.1)	40.4 (38.8, 42.1)	39.4 (37.6, 41.2)
Masculine gender: %	45.7 (44.4, 47.0)	55.6 (51.5, 59.6)	66.6 (61.8, 71.4)
Education: %			
Up to primary school	16.7 (11.9, 21.6)	28.8 (23.9, 33.7)	22.6 (17.0, 28.1)
High school/secondary	53.3 (47.8, 58.7)	52.6 (47.3, 57.9)	57.3 (50.0, 64.6)
College graduate	30.0 (21.1, 38.9)	18.6 (10.1, 27.1)	20.1 (10.6, 29.7)
Household income: %			
≤INR 10,000 (U.S. $200)	44.8 (34.4, 55.2)	61.4 (51.2, 71.5)	56.8 (46.0, 67.6)
INR 10,001–20,000	21.7 (18.7, 24.7)	18.9 (14.6, 23.2)	20.5 (13.0, 28.1)
≥INR 20,001 (U.S. $400)	32.7 (22.6, 42.8)	19.4 (11.1, 27.8)	22.2 (13.7, 30.6)
Declined to state income	0.8 (0.4, 1.1)	0.3 (<0.1, 0.7)	0.5 (<0.1, 1.2)
Sedentary lifestyle: %			
Sitting < 5 h/day	56.1 (52.1, 60.0)	54.5 (48.3, 60.7)	55.6 (47.1, 64.1)
Sitting ≥ 5 h/day	43.9 (40.0, 47.9)	45.5 (39.3, 51.7)	44.4 (35.9, 52.9)
Body mass index: mean kg/m^2^	25.7 (24.9, 26.4)	24.6 (23.8, 25.4)	24.6 (23.7, 25.6)
Unhealthy diet score: mean	10.0 (9.6, 10.4)	10.7 (10.1, 11.4)	12.2 (11.5, 13.0)
Systolic blood pressure: mean mm Hg	125.9 (123.9, 127.9)	125.1 (122.9, 127.3)	126.5 (123.6, 129.5)
Tobacco use: %			
Never smoker	79.5 (75.5, 83.5)	65.1 (61.2, 69.0)	62.3 (57.4, 67.3)
Former smoker (>6 months)	1.7 (1.3, 2.1)	2.0 (1.0, 3.0)	0.9 (0.2, 1.5)
Recent smoker (≤6 months)	18.8 (14.9, 22.7)	32.9 (29.0, 36.8)	36.8 (31.8, 41.7)
Diagnosed hyperlipidemia: %	2.3 (1.3, 3.2)	1.8 (0.6, 3.0)	1.7 (0.4, 2.9)
Diagnosed hypertension: %	15.5 (12.6, 18.5)	12.6 (9.2, 16.0)	9.7 (6.1, 13.3)
**KARACHI**			
Age: mean years	40.9 (39.9, 41.8)	41.9 (40.6, 43.2)	41.8 (40.0, 43.7)
Masculine gender: %	43.0 (41.1, 45.0)	49.4 (46.6, 52.2)	51.1 (46.4, 55.8)
Education: %			
Up to primary school	35.8 (30.9, 40.7)	27.0 (22.6, 31.5)	36.0 (25.5, 46.5)
High school/secondary	53.0 (49.4, 56.7)	55.5 (51.9, 59.0)	52.2 (44.4, 60.0)
College graduate	11.1 (7.9, 14.3)	17.5 (13.4, 21.5)	11.8 (7.0, 16.6)
Household income: %			
≤INR 10,000 (U.S. $200)	42.9 (39.1, 46.6)	34.6 (30.5, 38.8)	41.6 (33.3, 49.8)
INR 10,001–20,000	42.2 (39.6, 44.8)	43.5 (40.5, 46.5)	40.4 (34.7, 46.1)
≥INR 20,001 (U.S. $400)	14.2 (11.1, 17.3)	20.8 (16.8, 24.7)	16.5 (11.6, 21.4)
Declined to state income	0.7 (0.3, 1.1)	1.1 (0.6, 1.6)	1.5 (0.1, 3.0)
Sedentary lifestyle: %			
Sitting < 5 h/day	48.7 (44.9, 52.4)	51.9 (48.9, 55.0)	48.3 (43.6, 52.9)
Sitting ≥ 5 h/day	51.3 (47.6, 55.1)	48.1 (45.0, 51.1)	51.7 (47.1, 56.4)
Body mass index: mean kg/m^2^	25.4 (25.1, 25.7)	25.3 (24.9, 25.7)	25.0 (24.1, 25.9)
Unhealthy diet score: mean	8.3 (8.0, 8.6)	10.1 (9.8, 10.4)	10.1 (9.4, 10.8)
Systolic blood pressure: mean mm Hg	119.8 (118.6, 121.1)	121.4 (119.8, 123.0)	120.6 (118.3, 122.9)
Tobacco use: %			
Never smoker	72.7 (70.3, 75.1)	73.0 (69.3, 76.7)	59.2 (51.8, 66.6)
Former smoker (>6 months)	1.0 (0.5, 1.5)	1.7 (0.9, 2.5)	0.9 (0.1, 1.6)
Recent smoker (≤6 months)	26.3 (23.9, 28.7)	25.3 (21.8, 28.8)	39.9 (32.4, 47.4)
Diagnosed hyperlipidemia: %	2.3 (1.5, 3.1)	4.2 (3.1, 5.3)	5.1 (2.7, 7.4)
Diagnosed hypertension: %	17.3 (15.2, 19.4)	19.7 (17.2, 22.3)	16.8 (12.4, 21.2)

**Table 2:** Estimated population characteristics, by city and shellfish consumption. Survey estimation techniques were used to obtain estimates of population means and proportions. Here, the category “less than weekly” indicates that shellfish is consumed monthly or never. Disjoint categories within a column, within each city, sum to 100% (with rounding). Statistics are estimates for the characteristics of each city’s population of permanent residents age ≥20 years old, who are neither pregnant nor on bedrest. Population parameter estimates are based a large survey sample including participants from Karachi (unweighted *n* = 4017), Delhi (unweighted *n* = 5364), and Chennai (unweighted *n* = 6906).

Characteristic	Shellfish Consumption (Estimates and 95% Confidence Intervals)		
	Less than Weekly 74.8% (70.8%, 78.8%)	Weekly 13.9% (11.6%, 16.1%)	More than Weekly 11.3% (9.1%, 13.6%)
**CHENNAI**			
Age: mean years	40.8 (40.1, 41.6)	39.8 (38.8, 40.8)	38.9 (37.7, 40.1)
Masculine gender: %	45.0 (42.8, 47.2)	47.0 (44.4, 49.6)	44.7 (40.9, 48.6)
Education: %			
Up to primary school	15.7 (13.4, 17.9)	14.6 (11.2, 18.0)	17.7 (15.3, 20.2)
High school/secondary	71.6 (69.2, 74.0)	72.4 (66.7, 78.0)	71.2 (68.2, 74.3)
College graduate	12.8 (10.9, 14.7)	13.1 (9.7, 16.4)	11.0 (9.0, 13.1)
Household income: %			
≤INR 10,000 (U.S. $200)	82.1 (79.1, 85.1)	78.3 (74.1, 82.4)	83.6 (80.3, 86.9)
INR 10,001–20,000	12.6 (10.5, 14.8)	17.3 (12.9, 21.7)	12.1 (9.7, 14.6)
≥INR 20,001 (U.S. $400)	4.8 (2.9, 6.6)	4.0 (2.3, 5.6)	3.7 (2.0, 5.3)
Declined to state income	0.5 (0.2, 0.8)	0.5 (<0.1, 0.9)	0.6 (<0.1, 1.1)
Sedentary lifestyle: %			
Sitting < 5 h/day	25.5 (19.1, 31.9)	25.2 (18.7, 31.6)	20.0 (14.2, 25.8)
Sitting ≥ 5 h/day	74.5 (68.1, 80.9)	74.8 (68.4, 81.3)	80.0 (74.2, 85.8)
Body mass index: mean kg/m^2^	25.5 (25.2, 25.7)	25.5 (25.1, 25.9)	25.9 (25.5, 26.2)
Unhealthy diet score: mean	8.1 (7.9, 8.4)	10.5 (10.0, 10.9)	10.7 (10.0, 11.3)
Systolic blood pressure: mean mm Hg	121.2 (120.0, 122.3)	120.6 (118.4, 122.8)	120.4 (118.6, 122.2)
Tobacco use: %			
Never smoker	78.4 (76.3, 80.5)	78.1 (76.0, 80.2)	75.6 (72.7, 78.5)
Former smoker (>6 months)	2.3 (1.6, 3.1)	1.6 (0.9, 2.2)	1.6 (0.7, 2.5)
Recent smoker (≤6 months)	19.3 (17.1, 21.5)	20.3 (18.3, 22.4)	22.8 (19.9, 25.6)
Diagnosed hyperlipidemia: %	2.0 (1.4, 2.6)	1.2 (0.6, 1.9)	1.2 (0.5, 1.9)
Diagnosed hypertension: %	12.1 (10.8, 13.3)	9.0 (7.3, 10.7)	8.3 (5.6, 11.0)
**DELHI**			
Age: mean years	42.4 (40.6, 44.2)	42.0 (39.4, 44.7)	41.5 (39.3, 43.7)
Masculine gender: %	47.6 (46.8, 48.4)	90.1 (85.2, 95.1)	85.2 (79.8, 90.7)
Education: %			
Up to primary school	19.7 (14.7, 24.7)	13.4 (5.0, 21.9)	22.1 (13.6, 30.7)
High school/secondary	53.3 (48.4, 58.2)	58.7 (41.2, 76.1)	57.8 (43.1, 72.5)
College graduate	27.0 (18.2, 35.8)	27.9 (12.8, 43.0)	20.0 (7.4, 32.7)
Household income: %			
≤INR 10,000 (U.S. $200)	48.6 (38.4, 58.7)	61.3 (44.1, 78.6)	58.6 (47.8, 69.5)
INR 10,001–20,000	21.3 (18.1, 24.6)	11.7 (4.6, 18.8)	19.6 (10.9, 28.3)
≥INR 20,001 (U.S. $400)	29.5 (19.9, 39.1)	26.6 (12.1, 41.2)	20.1 (9.1, 31.2)
Declined to state income	0.6 (0.3, 1.0)	0.4 (<0.1, 1.1)	1.7 (<0.1, 3.8)
Sedentary lifestyle: %			
Sitting < 5 h/day	56.6 (52.5, 60.6)	37.2 (27.8, 46.5)	43.0 (28.5, 57.4)
Sitting ≥ 5 h/day	43.4 (39.4, 47.5)	62.8 (53.5, 72.2)	57.0 (42.6, 71.5)
Body mass index: mean kg/m^2^	25.4 (24.6, 26.2)	24.6 (22.9, 26.3)	25.4 (24.3, 26.5)
Unhealthy diet score: mean	10.2 (9.8, 10.6)	12.9 (12.2, 13.6)	13.8 (12.7, 14.9)
Systolic blood pressure: mean mm Hg	125.8 (123.9, 127.7)	123.5 (119.5, 127.5)	128.0 (122.3, 133.7)
Tobacco use: %			
Never smoker	76.3 (72.3, 80.4)	52.7 (39.9, 65.6)	50.4 (41.9, 59.0)
Former smoker (>6 months)	1.7 (1.3, 2.0)	0.8 (<0.1, 1.9)	2.1 (<0.1, 4.4)
Recent smoker (≤6 months)	22.0 (18.1, 26.0)	46.5 (33.5, 59.5)	47.5 (39.0, 56.0)
Diagnosed hyperlipidemia: %	2.1 (1.3, 3.0)	0.4 (<0.1, 1.3)	2.9 (0.1, 5.7)
Diagnosed hypertension: %	14.7 (11.9, 17.5)	12.2 (4.2, 20.1)	6.9 (2.9, 10.9)
**KARACHI**			
Age: mean years	41.3 (40.3, 42.3)	42.5 (40.2, 44.7)	42.3 (39.1, 45.5)
Masculine gender: %	45.8 (44.4, 47.1)	57.3 (51.0, 63.6)	51.9 (40.3, 63.6)
Education: %			
Up to primary school	32.1 (27.7, 36.4)	27.2 (17.1, 37.4)	49.2 (25.5, 72.9)
High school/secondary	54.4 (51.5, 57.3)	51.3 (43.3, 59.4)	44.9 (24.8, 64.9)
College graduate	13.6 (10.4, 16.7)	21.4 (14.5, 28.4)	6.0 (<0.1, 12.1)
Household income: %			
≤INR 10,000 (U.S. $200)	39.1 (35.6, 42.7)	33.3 (25.7, 40.9)	58.6 (46.0, 71.2)
INR 10,001–20,000	43.1 (40.8, 45.3)	39.0 (33.0, 45.1)	32.1 (23.9, 40.4)
≥INR 20,001 (U.S. $400)	16.9 (13.8, 19.9)	26.4 (18.5, 34.3)	7.3 (1.2, 13.4)
Declined to state income	0.9 (0.5, 1.3)	1.2 (<0.1, 3.0)	2.0 (<0.1, 5.0)
Sedentary lifestyle: %			
Sitting < 5 h/day	50.3 (47.6, 52.9)	47.3 (40.9, 53.6)	44.9 (36.4, 53.4)
Sitting ≥ 5 h/day	49.7 (47.1, 52.4)	52.7 (46.4, 59.1)	55.1 (46.6, 63.6)
Body mass index: mean kg/m^2^	25.4 (25.1, 25.7)	24.5 (23.6, 25.5)	24.3 (22.3, 26.3)
Unhealthy diet score: mean	9.2 (8.9, 9.4)	10.8 (9.9, 11.6)	9.6 (8.1, 11.0)
Systolic blood pressure: mean mm Hg	120.6 (119.5, 121.7)	121.3 (118.3, 124.3)	118.0 (113.1, 122.9)
Tobacco use: %			
Never smoker	72.4 (69.9, 74.9)	61.0 (52.7, 69.4)	53.2 (35.6, 70.7)
Former smoker (>6 months)	1.3 (0.9, 1.8)	0.8 (<0.1, 2.0)	1.3 (<0.1, 3.4)
Recent smoker (≤6 months)	26.3 (23.9, 28.7)	38.1 (29.7, 46.6)	45.5 (27.4, 63.6)
Diagnosed hyperlipidemia: %	3.5 (2.7, 4.3)	3.9 (1.6, 6.3)	0.5 (<0.1, 1.5)
Diagnosed hypertension: %	18.2 (16.3, 20.0)	22.5 (17.3, 27.6)	11.2 (4.9, 17.4)

**Table 3:** Odds ratios (95% confidence intervals) of prevalent diabetes according to fish or shellfish consumption, by city. Note: shellfish consumption is infrequent in Delhi and Karachi. Here, the category “less than weekly” indicates the food item is consumed monthly or never. Adjusted models controlled for age, age^2^, gender, body mass index class, waist–height ratio, sedentary lifestyle, educational attainment, tobacco use, tertile of unhealthy diet score, income, self-reported physician diagnosis of high blood pressure, and self-reported physician diagnosis of high cholesterol. Population parameter estimates are based on a large survey sample including participants from Karachi (unweighted *n* = 4017), Delhi (unweighted *n* = 5364), and Chennai (unweighted *n* = 6906).

Frequency of Consumption	Unadjusted			Adjusted Models		
	Chennai	Delhi	Karachi	Chennai	Delhi	Karachi
Fish Consumption						
Less than weekly	Reference	Reference	Reference	Reference	Reference	Reference
Weekly	0.73 (0.56, 0.95)	0.78 (0.64, 0.96)	1.04 (0.87, 1.24)	0.75 (0.56, 1.02)	0.95 (0.78, 1.14)	1.07 (0.87, 1.30)
More than weekly	0.72 (0.54, 0.96)	0.95 (0.72, 1.24)	1.22 (0.92, 1.61)	0.81 (0.61, 1.08)	1.18 (0.87, 1.58)	1.30 (0.94, 1.80)
Shellfish Consumption						
Less than Weekly	Reference	Reference	Reference	Reference	Reference	Reference
Weekly	0.95 (0.82, 1.09)	0.96 (0.59, 1.56)	1.22 (0.89, 1.68)	1.04 (0.87, 1.23)	1.03 (0.62, 1.69)	1.21 (0.84, 1.75)
More than Weekly	0.92 (0.78, 1.09)	1.23 (0.84, 1.81)	1.48 (0.94, 2.33)	1.08 (0.90, 1.30)	1.35 (0.90, 2.01)	1.68 (0.98, 2.86)

**Table 4:** Adjusted differences in mean HbA1c and fasting glucose (with 95% confidence intervals) according to fish or shellfish consumption, by city. Note: shellfish consumption is infrequent in Delhi and Karachi. Here, the category “less than weekly” indicates the food item is consumed monthly or never. Adjusted models controlled for age, age^2^, gender, body mass index class, waist–height ratio, sedentary lifestyle, educational attainment, tobacco use, tertile of unhealthy diet score, income, self-reported physician diagnosis of high blood pressure, and self-reported physician diagnosis of high cholesterol. Population parameter estimates are based on a large survey sample including participants from Karachi (unweighted *n* = 4017), Delhi (unweighted *n* = 5364), and Chennai (unweighted *n* = 6906).

Frequency of Consumption	HbA1c			Fasting Glucose		
	Chennai	Delhi	Karachi	Chennai	Delhi	Karachi
Fish Consumption						
Less than weekly	Reference	Reference	Reference	Reference	Reference	Reference
Weekly	−0.17 (−0.35, 0.02)	−0.09 (−0.22, 0.04)	0.00 (−0.11, 0.12)	−3.54 (−9.31, 2.22)	−1.05 (−3.96, 1.87)	1.46 (−1.55, 4.47)
More than weekly	−0.06 (−0.19, 0.07)	0.07 (−0.13, 0.28)	0.10 (−0.06, 0.26)	−1.29 (−5.58, 2.99)	4.56 (−0.60, 9.72)	5.00 (0.68, 9.32)
Shellfish Consumption						
Less than Weekly	Reference	Reference	Reference	Reference	Reference	Reference
Weekly	0.03 (−0.07, 0.13)	−0.04 (−0.34, 0.26)	0.05 (−0.15, 0.26)	0.13 (−2.77, 3.03)	−1.12 (−9.86, 7.63)	3.24 (−2.78, 9.27)
More than Weekly	0.11 (−0.02, 0.24)	0.05 (−0.22, 0.33)	0.14 (−0.12, 0.40)	2.15 (−1.69, 5.99)	1.14 (−7.06, 9.34)	6.08 (−2.71, 14.86)

## Supplementary Materials

The following are available online at https://www.mdpi.com/1660-4601/17/2/459/s1. Table S1: Sensitivity Analysis for Influence of Dietary Adjustment on Odds Ratios (95% Confidence Intervals) of Prevalent Diabetes According to Fish or Shellfish Consumption, by City. Table S2: Sensitivity Analysis for Influence of Dietary Adjustment on Differences in Fasting Glucose (95% Confidence Intervals) of Prevalent Diabetes According to Fish or Shellfish Consumption, by City. Table S3: Sensitivity Analysis for Influence of Dietary Adjustment on Differences in HbA1c (95% Confidence Intervals) of Prevalent Diabetes According to Fish or Shellfish Consumption, by City. Table S4: Sensitivity Analysis: Odds Ratios (95% Confidence Intervals) of Prevalent Diabetes According to Fish or Shellfish Consumption, by City, without Adjustment for Waist-Height Ratio. Table S5: Sensitivity Analysis: Adjusted Differences in Mean HbA1c and Fasting Glucose (with 95% Confidence Intervals) According to Fish or Shellfish Consumption, by City, without Adjustment for Waist-Height Ratio.
