# Supplementary material for: Potentially Heterogeneous Cross-Sectional Associations of Seafood Consumption with Diabetes and Glycemia in Urban South Asia
Source: Int J Environ Res Public Health. 2020 Jan 10;17(2):459. doi: 10.3390/ijerph17020459 (PMC7013926; doi:10.3390/ijerph17020459)
Supplement: Supplementary file 1 [file ijerph-17-00459-s001.pdf]

Supplemental Table 1. Sensitivity analysis for influence of dietary adjustment on odds ratios (95% confidence intervals) of prevalent diabetes according to fish or shellfish consumption, by city. In the "models unadjusted for diet", models controlled for age, age^2, gender, BMI class, waist–height ratio, sedentary lifestyle, educational attainment, tobacco use, income, self-reported physician diagnosis of high blood pressure, and self-reported physician diagnosis of high cholesterol. In the models "adjusted for consumption of fruits and vegetables", models were further adjusted for those two dietary items. Population parameter estimates are based on a large survey sample including participants from Karachi (unweighted n = 4017), Delhi (unweighted n = 5364), and Chennai (unweighted n = 6906).

	Unadjusted for Diet			*Adjusted for Consumption of Fruits and Vegetables*		
	**Chennai**	**Delhi**	**Karachi**	Chennai	Delhi	Karachi
Fish Consumption						
Less than weekly	Reference	Reference	Reference	Reference	Reference	Reference
Weekly	0.75 (0.56, 1.02)	0.95 (0.78, 1.14)	0.99 (0.82, 1.20)	0.75 (0.56, 1.00)	0.95 (0.79, 1.15)	1.01 (0.84, 1.23)
More than weekly	0.81 (0.61, 1.07)	1.18 (0.88, 1.58)	1.21 (0.87, 1.68)	0.81 (0.61, 1.07)	1.19 (0.89, 1.59)	1.24 (0.90, 1.72)
Shellfish Consumption						
Less than Weekly	Reference	Reference	Reference	Reference	Reference	Reference
Weekly	1.02 (0.87, 1.19)	1.03 (0.62, 1.69)	1.17 (0.80, 1.70)	1.02 (0.87, 1.20)	1.04 (0.63, 1.71)	1.19 (0.82, 1.72)
More than Weekly	1.06 (0.88, 1.27)	1.35 (0.91, 2.01)	1.66 (0.97, 2.84)	1.08 (0.90, 1.30)	1.37 (0.91, 2.05)	1.66 (0.97, 2.85)

Supplemental Table 2. Sensitivity analysis for influence of dietary adjustment on differences in fasting glucose (95% confidence intervals) of prevalent diabetes according to fish or shellfish consumption, by city. In the "models unadjusted for diet", models controlled for age, age^2, gender, BMI class, waist–height ratio, sedentary lifestyle, educational attainment, tobacco use, income, self-reported physician diagnosis of high blood pressure, and self-reported physician diagnosis of high cholesterol. In the models "adjusted for consumption of fruits and vegetables", models were further adjusted for those two dietary items. Population parameter estimates are based on a large survey sample including participants from Karachi (unweighted n = 4017), Delhi (unweighted n = 5364), and Chennai (unweighted n = 6906).

	Unadjusted for Diet			*Adjusted for Fruits, Vegetables, Fried Foods, Desserts*		
	**Chennai**	**Delhi**	**Karachi**	Chennai	Delhi	Karachi
Fish Consumption						
Less than weekly	Reference	Reference	Reference	Reference	Reference	Reference
Weekly	−3.62 (−9.42, 2.19)	−1.20 (−4.18, 1.78)	0.70 (−2.26, 3.66)	−3.77 (−9.36, 1.83)	−1.04 (−4.05, 1.97)	0.88 (−2.09, 3.85)
More than weekly	−1.42 (−5.76, 2.92)	4.15 (−1.17, 9.47)	4.25 (−0.06, 8.55)	−1.47 (−5.75, 2.82)	4.66 (−0.66, 9.98)	4.46 (0.14, 8.79)
Shellfish Consumption						
Less than Weekly	Reference	Reference	Reference	Reference	Reference	Reference
Weekly	−0.45 (−3.05, 2.15)	−1.73 (−10.51, 7.04)	2.94 (−3.13, 9.00)	−0.32 (−2.94, 2.31)	−1.68 (−10.54, 7.19)	3.01 (−3.05, 9.07)
More than Weekly	1.61 (−2.12, 5.34)	0.47 (−7.26, 8.19)	5.96 (−2.86, 14.77)	2.05 (−1.66, 5.76)	1.20 (−6.66, 9.06)	6.03 (−2.78, 14.84)

Supplemental Table 3. Sensitivity analysis for influence of dietary adjustment on differences in HbA1c (95% confidence intervals) of prevalent diabetes according to fish or shellfish consumption, by city. In the "models unadjusted for diet", models controlled for age, age^2, gender, BMI class, waist–height ratio, sedentary lifestyle, educational attainment, tobacco use, income, self-reported physician diagnosis of high blood pressure, and self-reported physician diagnosis of high cholesterol. In the models "adjusted for consumption of fruits and vegetables", models were further adjusted for those two dietary items. Population parameter estimates are based on a large survey sample including participants from Karachi (unweighted n = 4017), Delhi (unweighted n = 5364), and Chennai (unweighted n = 6906).

	Unadjusted for Diet			*Adjusted for Consumption of Fruits and Vegetables*		
	**Chennai**	**Delhi**	**Karachi**	Chennai	Delhi	Karachi
Fish Consumption						
Less than weekly	Reference	Reference	Reference	Reference	Reference	Reference
Weekly	−0.17 (−0.36, 0.02)	−0.09 (−0.22, 0.04)	−0.02 (−0.14, 0.09)	−0.18 (−0.36, 0.00)	−0.09 (−0.22, 0.04)	−0.02 (−0.13, 0.10)
More than weekly	−0.07 (−0.20, 0.07)	0.07 (−0.14, 0.28)	0.08 (−0.08, 0.24)	−0.07 (−0.21, 0.06)	0.08 (−0.12, 0.29)	0.09 (−0.07, 0.24))
Shellfish Consumption						
Less than Weekly	Reference	Reference	Reference	Reference	Reference	Reference
Weekly	0.00 (−0.08, 0.09)	−0.05 (−0.35, 0.25)	0.04 (−0.17, 0.25)	0.01 (−0.08, 0.10)	−0.05 (−0.35, 0.26)	0.04 (−0.16, 0.25)
More than Weekly	0.09 (−0.03, 0.21)	0.04 (−0.22, 0.30)	0.14 (−0.13, 0.40)	0.10 (−0.02, 0.22)	0.06 (−0.21, 0.33)	0.14 (−0.13, 0.40)

Supplemental Table 4. Sensitivity analysis: odds ratios (95% confidence intervals) of prevalent diabetes according to fish or shellfish consumption, by city, without adjustment for waist–height ratio. Note: shellfish consumption is infrequent in Delhi and Karachi. Here, the category "less than weekly" indicates the food item is consumed monthly or never. Adjusted models controlled for age, age^2, gender, BMI class, sedentary lifestyle, educational attainment, tobacco use, tertile of unhealthy diet score, income, self-reported physician diagnosis of high blood pressure, and self-reported physician diagnosis of high cholesterol. Population parameter estimates are based on a large survey sample including participants from Karachi (unweighted n = 4017), Delhi (unweighted n = 5364), and Chennai (unweighted n = 6906).

	Models Without Waist-Height Ratio Adjustment		
	**Chennai**	Delhi	Karachi
Fish Consumption			
Less than weekly	Reference	Reference	Reference
Weekly	0.76 (0.56, 1.03)	0.96 (0.80, 1.16)	1.07 (0.88, 1.31)
More than weekly	0.80 (0.60, 1.08)	1.18 (0.88, 1.57)	1.32 (0.95, 1.82)
Shellfish Consumption			
Less than Weekly	Reference	Reference	Reference
Weekly	1.03 (0.87, 1.22)	0.98 (0.60, 1.60)	1.23 (0.86, 1.76)
More than Weekly	1.06 (0.88, 1.27)	1.27 (0.85, 1.90)	1.64 (0.98, 2.77)

Supplemental Table 5. Sensitivity analysis: adjusted differences in mean HbA1c and fasting glucose (with 95% confidence intervals) according to fish or shellfish consumption, by city, without adjustment for waist–height ratio. Note: shellfish consumption is infrequent in Delhi and Karachi. Here, the category "less than weekly" indicates the food item is consumed monthly or never. Adjusted models controlled for age, age^2, gender, BMI class, sedentary lifestyle, educational attainment, tobacco use, tertile of unhealthy diet score, income, self-reported physician diagnosis of high blood pressure, and self-reported physician diagnosis of high cholesterol. Population parameter estimates are based on a large survey sample including participants from Karachi (unweighted n = 4017), Delhi (unweighted n = 5364), and Chennai (unweighted n = 6906).

	HbA1c			Fasting Glucose		
	**Chennai**	**Delhi**	**Karachi**	Chennai	Delhi	Karachi
Fish Consumption						
Less than weekly	Reference	Reference	Reference	Reference	Reference	Reference
Weekly	−0.17 (−0.35, 0.02)	−0.08 (−0.21, 0.05)	0.01 (−0.11, 0.12)	−3.53 (−9.30, 2.24)	−0.82 (−3.82, 2.17)	1.54 (−1.54, 4.61)
More than weekly	−0.07 (−0.21, 0.07)	0.08 (−0.13, 0.28)	0.11 (−0.05, 0.27)	−1.47 (−5.93, 2.99)	4.61 (−0.49, 9.71)	5.12 (0.79, 9.46)
Shellfish Consumption						
Less than Weekly	Reference	Reference	Reference	Reference	Reference	Reference
Weekly	0.02 (−0.08, 0.12)	−0.07 (−0.37, 0.24)	0.06 (−0.15, 0.26)	−0.02 (−2.95, 2.91)	−1.82 (−10.52, 6.87)	3.39 (−2.68, 9.46)
More than Weekly	0.10 (−0.03, 0.22)	0.03 (−0.25, 0.30)	0.13 (−0.13, 0.39)	1.66 (−2.04, 5.36)	0.37 (−7.63, 8.37)	5.83 (−2.89, 14.54)
